# Community Health Workers' and Pharmacists' Perspectives of a CHW‐Pharmacist Collaboration Model to Support Medication Adherence

**DOI:** 10.1111/hex.70630

**Published:** 2026-03-16

**Authors:** Carole Bandiera, Sabuj Kanti Mistry, Elizabeth Harris, Mark F. Harris, Parisa Aslani

**Affiliations:** ^1^ Sydney Pharmacy School, Faculty of Medicine and Health University of Sydney Sydney Australia; ^2^ School of Population Health, University of New South Wales Sydney Australia; ^3^ International Centre for Future Health Systems, University of New South Wales Sydney Australia

**Keywords:** collaboration model, collaborative practice, community health workers, health navigators, interprofessional collaboration, medication adherence, pharmacists

## Abstract

**Introduction:**

Interprofessional collaboration is key in supporting patient medication adherence. Community health workers (CHWs) can bridge the gap between community and health services and can collaborate with pharmacists to support medication adherence. A collaborative CHW‐pharmacist practice model was developed in the United States of America (USA), where CHWs and pharmacists collaborate to develop an action plan to address medication adherence barriers, and provide follow‐up with patients. The present study aimed to investigate (1) opinions of pharmacists and CHWs working in Australia and New Zealand about this model, and (2) how the model could be implemented in their respective country.

**Method:**

Semi‐structured interviews were conducted with CHWs and pharmacists working in Australia or New Zealand. Questions addressed the CHWs' and pharmacists' perspectives on the CHW‐pharmacist collaborative practice model. Interviews were audio‐recorded, transcribed verbatim, and coded using a thematically inductive process.

**Results:**

Twenty‐nine participants (16 pharmacists and 13 CHWs) were interviewed, 19 worked in Australia and 10 in New Zealand. Participants' opinions about the model were categorised into three themes: (1) perceptions of the model, (2) challenges and (3) facilitators to the implementation of the model. Most participants recognised the model's potential to support medication adherence and appreciated the role of CHWs in bridging the cultural gap with patients. Reported challenges to model implementation included concern about the overlap of services with existing pharmacy services, cultural considerations, CHWs' limited clinical training, need for resources and sharing information with the healthcare team members. Facilitators involved clarification of roles, training for CHWs, and fostering collaboration with the other members of the healthcare team.

**Conclusion:**

While the CHW‐pharmacist collaborative practice model was seen as valuable to support medication adherence in Australia and New Zealand, challenges were highlighted to be considered before implementation. Clear definition of roles and guidelines on collaborative practices to support medication adherence may facilitate the effective implementation of the model.

**Patient or Public Contribution:**

Patients, service users, care‐givers, people with lived experience or members of the public were not involved in the study design or conduct of study, analysis or interpretation of the data or in preparation of the manuscript.

## 1 Introduction

Medication adherence is the process by which patients take their prescribed medication. Patients' medication taking can be characterised by three interrelated phases: (1) initiation (first dose taken), (2) implementation (daily dosing), and (3) discontinuation (premature stopping of treatment) [1]. About one‐third of patients never fill their first prescription [2], and of those who do, up to 50% do no take their medications as prescribed and only less than 50% of these patients are persistent with medication taking 2 years after their initial prescription [2]. Nonadherence is a silent pandemic that leads to poor health outcomes, poor quality of life, mortality, and increased health care costs [2, 3, 4, 5, 6]. The complexity of patients' medication adherence behaviours is influenced by numerous factors, including determinants related to the patients themselves (e.g., personal beliefs), the disease (e.g., symptoms), the treatment (e.g., adverse effects), the social and economic environment (e.g., food insecurity), healthcare professionals, and the health system (e.g., patients' out of pocket costs for medications) [7, 8, 9]. An interprofessional healthcare team, with two or more healthcare professionals working together [10] can identify medication adherence issues, share information, evaluate, address, support and monitor medication adherence to optimise medication management and adherence [11].

Community health workers (CHWs) are frontline public health workers, often sharing the same cultural background as the communities they serve [12]. They bridge the gap between health and social services and the community – mostly socially disadvantaged populations who experience barriers to accessing care, have an increased risk of developing health issues, and face health disparities and inequities, associated with social, economic or environmental factors [13, 14]. For instance, health disparities are faced by Aboriginal and Torres Strait Islander peoples in Australia [15, 16] and Māori in New Zealand [17, 18]. CHWs deliver services in a culturally adapted way, promoting access improvement and uptake of healthcare services. They provide health education, collect health data, facilitate the relationship between the healthcare team and the patient, deliver treatment or clinical care and provide psychosocial support [12, 19]. CHWs' primary healthcare services have been effective in improving maternal, newborn and child health, mental health, sexual and reproductive health, and play an important role in the prevention, diagnosis, treatment and care of communicable and noncommunicable diseases [20]. Additionally, CHWs have a key role to play to support medication adherence in underserved populations [21, 22, 23].

The integration of CHWs within the healthcare team could support other healthcare professionals to improve patient health outcomes and adherence [24, 25]. While there is a growing evidence of the collaboration between physicians, nurses and pharmacists to support adherence [26, 27, 28, 29], the research on the collaboration between CHWs and pharmacists, to improve adherence, is limited [23, 30].

Pharmacists may experience challenges in identifying medication adherence barriers, such as those that are regarded as social determinants of health (e.g., food or housing insecurity), especially in vulnerable and underserved patients [31, 32]. Working in collaboration with CHWs seems promising as CHWs can improve patient referral to pharmacists so that more patients can benefit from pharmaceutical services [30]. In a recent systematic review [23], eight studies were identified that reported how pharmacists and CHWs can collaborate to support medication adherence, with three studies [33, 34, 35] showing a significant improvement in medication adherence.

**Figure 1:**
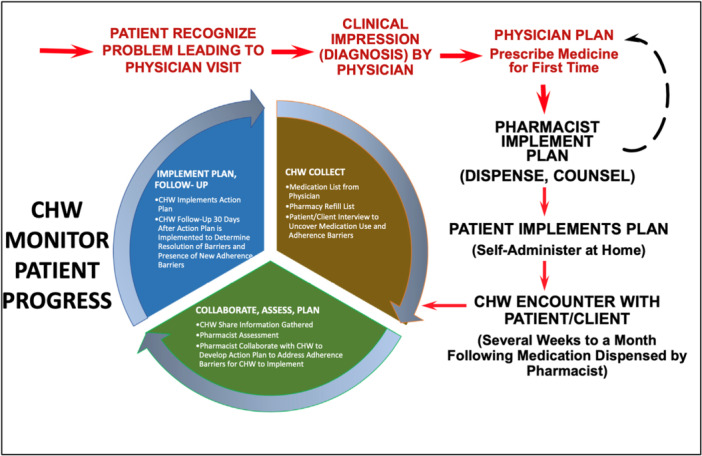
Collaborative CHW‐pharmacist practice model discussed with participants in our qualitative study, published by Segal R, Angaran DM, Odedina FT, Zeigler ML, Wallace JL, Opportunities and responsibilities for pharmacists to improve their effectiveness in addressing medication adherence through culturally sensitive collaborations with community health workers. J Am Pharm Assoc (2003). 2020 Jul‐Aug;60(4):e25‐e30. doi: 10.1016/j. japh.2020.02.023 [25]. Reproduction of the figure in this paper with permission from Elsevier. CHW, community health worker.

We have identified only one well‐defined model of CHW‐pharmacist collaborative practice in the literature. Segal et al. proposed a collaborative CHW‐pharmacist practice model where pharmacists and CHWs work closely together to support patient medication adherence [25] (Figure 1 published by Segal et al. [25], reproduced in this article with permission from Elsevier). In this model, the CHW conducts an interview with the patient to understand their medication use, adherence and any barriers to medication adherence. This interview is conducted several weeks after the patient has implemented their medication taking plan at home and after they have been counselled by a pharmacist when collecting their medication following the physician's prescription [25]. The CHW then shares their finding with the pharmacist for assessment, and the pharmacist develops an action plan to address medication adherence barriers that the CHW can implement with the patient [25]. The CHW then follows‐up resolution of adherence barriers and presence of new ones with the patient [25]. This collaborative practice model was developed by a research team in the United States of America (USA) and may need tailoring to be relevant to the context of the Australian and New Zealand health systems. To the best of our knowledge, such models do not exist in Australia and New Zealand considering that there are CHWs collaborating with other healthcare professionals in these two countries.

The present study investigated CHWs' and pharmacists' opinions about the CHW‐pharmacist collaborative practice model developped in the USA, as a first step towards adapting and implementing the model in the context of Australia and New Zealand. The research questions were: 1) What do the participants think about this collaborative practice model?, and 2) How do participants think this model would work in Australia (or New Zealand, as relevant)?

## 2 Methods

The methods for this qualitative study have been reported in more detail elsewhere [36]. The study was conducted in accordance with the declaration of Helsinki and was approved in June 2024 by the Human Research Ethics Committee of The University of Sydney (2024/HE000334). We followed the standards for reporting qualitative research [37] and the consolidated criteria for reporting qualitative research (COREQ) checklist was completed (Supporting Information S1: File 1) [38].

### 2.1 Recruitment of Participants

Participants were eligible to participate if they (1) were a CHW, a CHW supervisor or a pharmacist who has been working in health services for at least 6 months in Australia or New Zealand, and (2) spoke English and did not need an interpreter to participate. The study was mostly promoted through the researchers' professional networks, both in Australia and New Zealand. It was also advertised through newsletters sent to pharmacists in Australia, on the researchers' personal LinkedIn account (LinkedIn Corporation) and on closed pharmacist groups on Facebook (Meta Platforms Inc.).

Informed consent was obtained from the participants before data collection. All study forms (e.g., participant consent form, demographic data and receipt of the electronic voucher) were completed by participants in hard copy or online – using Research Electronic Data Capture (RedCap, Vanderbilt University), a secure, web‐based software platform designed to support data capture for research studies [39].

### 2.2 Data Collection

The in‐depth interviews were conducted by CB either online (using Zoom Video Communications Inc., or Teams, Microsoft Corporation) or in‐person, from July 2024 to February 2025. Two interview guides were developed to address the overall study aims: one for pharmacists and one for CHWs (Supporting Information S2 and S3: Files 2 and 3), and were piloted. The questions were open‐ended to ensure that participants were able to provide as much information as possible, and prompts were used to gain further information, if needed.

The interview guides explored (1) participants' professional background, (2) perceived roles of pharmacists/CHWs, (3) interprofessional collaborations with pharmacists/CHWs, (4) discussion of the published collaborative CHW‐pharmacist practice model [25], (5) barriers to the interprofessional collaboration, (6) facilitators to the interprofessional collaboration, and (7) perspectives on the CHW‐pharmacist collaboration in practice for the future.

This paper focused on the themes identified in the part (4), exploring participants' opinions about the published collaborative CHW‐pharmacist practice model in addressing medication adherence [25]. The results from the other sections have been published elsewhere [36].

The model presented in Figure 1 was shown to the participants, and questions from the interview guides, which focused on exploring this model, were: (1) What do you think about it?, and (2) How do you think this model would work in Australia (or New Zealand, as relevant)?

Data collection was stopped when data saturation was reached, defined as when no new codes or themes were generated by the researchers in at least two consecutive interviews [40]. Demographic characteristics of the participants were collected before the interview on gender, age, length of work experience in health, and cultural background.

The interviews were audio‐recorded and transcribed verbatim. All participants were invited to review their interview transcripts; two pharmacists and seven CHWs reviewed it. Only one participant made minor changes that did not impact the analysis. Participants were reimbursed with electronic vouchers to spend in a local grocery store (AUD 100 or NZD 120 for pharmacists and AUD 75 or NZD 90 for CHWs).

### 2.3 Data Analysis

The transcripts were de‐identified and analysed following Braun and Clarke's work through a reflexive thematic analysis and an inductive process, acknowledging the researcher's subjectivity [41, 42, 43]. The interviews were coded in chronological order, which allowed for the iterative development of the coding tree that combined findings from both pharmacists and CHWs. Ideas, perspectives and viewpoints from both cohorts were integrated in the coding tree and analysed. Codes were refined iteratively after each interview was coded to produce the final common set of themes and subthemes.

CB, who conducted the reflexive thematic analysis, is a pharmacist by training, which might have influenced the interpretation of the findings towards a better understanding of the perceptions, challenges and facilitators for pharmacists within the CHW‐pharmacist collaboration. The coding tree was discussed and reviewed until consensus achieved with SKM, who is not a pharmacist by training, and PA. The codes were then reviewed by EH and MH who are not pharmacists by training. All researchers were trained, and have extensive experience, in qualitative research.

The interviews were coded using the qualitative data analysis software NVivo version 14 (QSR International Pty Ltd). The findings have been described narratively and illustrated with relevant quotes.

## 3 Results

### 3.1 Included Participants

In total, 29 participants were interviewed (16 pharmacists and 13 CHWs). Demographic data are presented in Supporting Information S4: File 4.

After advertising the study on the professional platform LinkedIn (LinkedIn Corporation), three interviews were conducted with fraudulent participants, falsely claiming to be pharmacists or CHWs [44]. The data collected from these three participants were not analysed.

Most pharmacists interviewed were hospital pharmacists (5/16) and some community pharmacists had combined roles with academic, hospital or project‐based positions (7/16). There was a range of CHW positions, e.g., health navigator (4/13), cultural support worker (2/13) or bilingual community educator (2/13). Participants were mostly female (10/16 pharmacists and 10/13 CHWs). Median years of work experience was 9 years (interquartile range (IQR) 6–19) for pharmacists and 8 years (IQR 3–17) for CHWs. There were 12 pharmacists and 7 CHWs interviewed who worked in Australia, and 4 pharmacists and 6 CHWs who worked in New Zealand.

Eleven interviews with pharmacists (69%) were online, 3 were face to face and 2 by phone. Five interviews with CHWs (38%) were online, 6 face to face and 2 by phone. The median duration of the interviews was 30 min (IQR 26‐34) with pharmacists and 31 min (IQR 27‐36) with CHWs.

Data saturation (i.e., when no new codes or themes were generated by the researchers) was reached after 12/16 interviews with pharmacists and 10/13 interviews with CHWs; further interviews were conducted to confirm data saturation.

**Figure 2:**
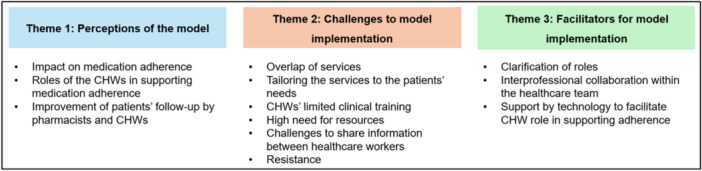
Key themes and subthemes.

Participants expressed their opinions about the CHW‐pharmacist collaborative practice model around three main themes: (1) perceptions of the model, (2) challenges to model implementation, and (3) facilitators for model implementation. Figure 2 summarises the key themes and subthemes.

Relevant quotes, along with their unique participant identifier, are presented in Table 1.

**Table 1:** Quotes illustrating the themes and subthemes (N/A = not applicable)

Themes and subthemes	Pharmacists' quotes	CHWs' quotes
**Theme 1: Perceptions of the model**		
Impact on medication adherence	1.1.‘I think that that would be good. It's particularly in matters of adherence. I think this is one of the major opportunities, really… pharmacists haven't quite cracked the nut of supporting patients adherence in a really meaningful and tailored way. (…) So, when you're not actually coming back to the patient's home, that you're not able to check on that.’ P14 1.2.‘I think that kind of thing would be great because, probably the most vulnerable patients are the ones that don't really understand their medicines. Don't necessarily understand the importance of them, or, (…) may have some cognitive issues, that mean medication compliance and stuff like that can be an issue.’ P10	1.3.‘There's an awful lot of people with a lot of old medication that they've never taken (…), and especially, they'll take it until they feel better, like antibiotics, and then they'll save them till next time. They don't understand.’ P28 1.4.‘I think this is a very informative concept that can implement in Australia because what I see like people who are aged, aged people benefit these types of support actually. (…) But I think if you present Community Health Navigator come every week and then see all the medication and things. I think it will be really great and helpful for these types of patients. People who forgot that take their medication.’ P02
Roles of the CHWs in supporting medication adherence	2.1.‘when a doctor is charting a medication for the first time (…), but a community healthcare worker is there, they might be able to kind of filter and synthesise that information and then help the patient understand it better. (…) And that community healthcare worker might be, you know, that third voice to explain that to the patient. So I think there is potential there. (…) If they [CHWs] were taking the patient to the GP appointment or to the pharmacist visit where they could help, you know the patient understand them and their about their medications, then that might be a good thing as well.’ P18	2.2. ‘I think it will definitely work because that two things: BCN [bilingual community navigator] are… normally they speak the same language. (…) people with the literacy issue, they just come up with so many concerns and things they have, sometimes they like to share that, but there's nobody available to share that. Even families, everyone is busy. (…) So being very busy and taking one hour will be great, because they will pull out lots of information. Also, from the psychology background, it's always good. And also pharmacists, they don't have time to give attention to so many community members. So BCN would be really good because they are like a connecting channel. Bridging the Gap, you can say that.’ P04
Improvement of patients' follow‐up by pharmacists and CHWs	3.1. ‘Yes definitely it will help. Because sometimes, for example, if I don't have time to personally go to the patient myself, for very far away patient, I may not see the patient until, like, in three weeks. By that time, you know, it's already too late. So in that case, maybe they [CHWs] can help to step in and until I can see the patient to see if there's anything urgent. If it's urgent, I can then, you know, call the doctor. I feel they can definitely help in some ways, but just not, like, 100%. I think we could definitely work collaboratively.’ P15	N/A
**Theme 2: Challenges to model implementation**		
Overlap of services	4.1. ‘I wonder, how would go with because we already have HMRs,^a^ and we have MedsCheck^b^ and everything in Australia where this would slot in. (…) So I can see pros and cons. I think it's good that you have a healthcare worker in this process, because I think in Australia, for HMR, you can have your healthcare worker there with you. And if a pharmacist is asking questions, and if you're unsure, the healthcare worker could be like, ‘This is what it means’. So from that regard, I think because we already have, in a way, systems like that. I'm not sure in the United States if they have HMRs or MedsCheck, so I'm not sure how that was sort of the system. I'm not against. I think we think anything to help the process would be better. It's just, I wonder how we're slot into the Australian system when you can already have healthcare workers with a patient during a HMR. (…) If you have people overtaking roles and everything, it can kind of muddy the waters about who did what and everything there.’ P32	4.2. ‘Yeah, I'm sure it could. Yeah, I'm actually sure that could and there are other health workers, community health support workers who are in the homes of some of these people more regularly than we are, like the people that oversee the Medicine oversight. (…) they have a report above them who is an RN [registered nurse], so if they've got any issues, they would report them back to the RN. (…) I would assume, if they were worried about the medication, would either go back to the GP or to the pharmacist directly, but probably more to the GP.’ P28
Tailoring the services to the patients' needs	5.1.‘I think the challenge would be to apply it to just cases where it needed sorting out. I mean, there's people who are anxious about their medication because it's but complicated. They don't necessarily need to see a pharmacist on a regular basis. They certainly would when there's any change of medication or any new medication.’ P27	5.2. ‘(…) I know that in the Pacific culture, (…) let's say the patient is elderly, (…) or be like a grandchild, they will look after the medications for the elderly. So I think implementing understanding some cultural barriers to why these not like, people in cold backgrounds don't take medication or adhere to medication. I think there needs to be more worked. There needs to be more research around that before implementing like a model like that. So, yeah, but I can see, I think it can work, but we just got, I think it's just a trial, so gotta trial that out.’ P17 5.3. ‘Absolutely great. But (…) it would be very challenging for Australia. The reason I say that is, I say the outcome is the community heath navigator evolve, it will be really helped the clients for the long term. But like, for example, my role working for the XX Local Health District is a short term, they usually maximum follow up with the clients standard is maximum three months unless it's a critical reason why keep longer.’ P03
CHWs' limited clinical training	6.1. ‘You have to reference what a fever is not like, because otherwise, if you put too much pressure on that healthcare worker, and they have to make too much of a judgement themselves, that's the grey area. (…) you don't want them to feel like they're taking on more than they should be. (…) Sometimes feel that people like that [CHWs] are put in a position where they expected to make a clinical judgement, which is not their decision. (…) I don't think they should be pressured to work beyond their scope.’ P24 6.2. ‘I guess my only concern is health navigators generally aren't a registered workforce. So I guess, what kind of health information do they need? What do they adhere to, code of conduct, or who do they report to, or do they have, like a safety mechanism, know when to seek help, you know these kind of things that health professionals would already know. (…) Just making sure that they know their scope (…) It would be really good to gather that information, but then making sure they know that they shouldn't be advising on how to manage those side effects, for example.’ P29 6.3. ‘I just feel like there's, they can't go out there to check all the side effects. They can have, you know, a questionnaire or a health assessment form, they can go through and tick, tick all the boxes or then… whether the patient is compliant, whether they're having any side effects or and check other symptoms, like pain or whatever, constipation and all that. But what else can they offer after that? Because that's still limited to what they can do.’ P06	6.4. ‘You can only remind them [the patients] ‘Now it's time for you to take medication’, but you cannot administer the medication. So I think in Australia, RN [register nurses] can do so, but you know, RN require (…) four year learning to get a degree. So it's much higher requirement for the person who can administer the medication… they must have the medical background, but the support worker they have the background of providing help, like very practical help. For example, when you walk, I support you so you will not fail. (…) Those kind of help, but (…)… not specialised in medication, not much related to the health or medication. They have a little bit background, (…) they are not trained in that high level. (…) So you just mentioned that example, it really require the support worker have the medical background and also maybe have the language level to say, to pass on the information and also interpret from the pharmacist. I think if, if a person have the… can be a RN and also can be a NAATI [National Accreditation Authority for Translators and Interpreters] certified interpreter, he will not be a support worker.’ P08 6.5. ‘See, the navigators have to be mindful that they're not health professionals in the same way as a pharmacist or a practice nurse or a doctor, but nonetheless, they've got quite a bit of insight into patients individual situation at a day to day level.’ P27 6.6. ‘Potentially, maybe not within our role, because we don't have an understanding of medication, that's not something we are generally involved with.’ P25
High need for resources	7.1. ‘It would require pharmacists to be, I guess, available for it. So I think it would have to be something that would be almost a role for a pharmacist. I think if, like, for instance, if it was my position right now on the ward, I don't think I would necessarily have time to be able to do enough follow up with all the patients that do go home with community health workers. If there was a role that was available for a pharmacist, let's say full time, or a couple of days a week to have the time to properly do it, then I think it would 100% work. I think it would actually be quite, quite handy, and probably take a lot of stress off the doctors and GPs as well, really.’ P12 7.2. ‘I just realised it uses a lot of resource. (…) Not in the foreseeable future [the model cannot be implemented in Australia]. I don't think the fundings would allow… (…) With the inflation and economy right now, like, how much would you need to pay a patient, community health worker per hour to do this? (…) I am just thinking of the cost side of things because you got to travel to the patient's home and look at them, and then come back.’ P06	7.3. ‘I think for us, that would be a lot of work to add that to what we already do. I think it's a really good idea, potentially, if someone… we have district nurses that go out and see people, so you know that they are another set of eyes for our clients, but I think it would increase our workload, I think, and you know, we're not maybe able to have time doing our core role if we just looked at medication.’ P25
Challenges to share information between healthcare workers	8.1. ‘I'm also thinking, so the privacy part of it, I'm not sure. When it comes to the community health worker under the privacy at which part they will fall under, how much the pharmacist can release those information.’ P13 8.2. ‘I think one of the challenges for this would be making sure that… in terms of electronic medication lists and how they get transferred from a physician to a community pharmacy. Oftentimes, a GP and a pharmacy will have quite unique systems that don't talk to each other, and unless the patient is signed up for, say, something like a My Health Record^c^, then it might be a challenge as well. (…) even some patients don't sign up for the My Health Record. And so I think in those instances, that's where a community healthcare worker might play a more even important role in making sure that everyone across the healthcare spectrum knows the patient's medications, including the patients’ P18 8.3. ‘I think too often there's communication between the doctor and the community health worker, not only via the pharmacist about medicines you know, I think that's an important part of this, that might bypass the pharmacist. But yeah, other than that, (…) that's how it works pretty much.’ P01	8.4. ‘The second issue is this community worker to… from the Home Care Package, they usually don't contact the pharmacies at all. They not aware what's the current medication list for the clients. And they usually just pick up the Webster‐pak^d^ from the pharmacies and just give it to the patient. That's it. (…) compliance yes, they can follow up or any medication issues, or like side affections, or even like me to speak to GP or pharmacist. None of them doing that. Even we promote it. I think that will be challenge. If… They probably would say it's not their role, whatever.’ P03
Resistance	9.1. ‘I think that my main takeaway from it that there are good things to having it [the practice model] there. (…) but I could see it being a bit cumbersome in the healthcare system.’ (Investigator: Yeah. And how would it be annoying, or what would be the…?) (…) I think it'd be more annoying from the pharmacists perspective, (…) if you're having a healthcare worker go out and kind of do, in essence, a HMR, a mini HMR, and connect to the pharmacist, the pharmacist so just like ‘I could have just done that myself’. So (…) I think that would be a barrier, versus the pharmacist going there and you have a healthcare worker already there, facilitating and helping and making easier.’ P32	9.2. ‘Other clinicians, let's say, like doctors and stuff, they may feel a bit of, there might be a bit of resistance, you know, because I know as a health coach, there was a bit of resistance with the doctors I worked with, but then eventually they kind of understood my role. So I think it's just introducing to these clinicians and staff, the purpose of the model and what we're looking to establish and improve patient outcomes.’ P17
**Theme 3: Facilitators for model implementation**		
Clarification of roles	10.1. ‘If you have people overtaking roles and everything, it can kind of muddy the waters about who did what and everything there. And I think it's like so I think having clear, defined roles in that perspective would make it better.’ P32 10.2. ‘It could work, but I feel that it has to be under tight like supervision, by the health professionals until they are very well trained, you know, to do all everything by themselves.’ P15	10.3. ‘I don't want to be so… getting involved with people's medication, I think could be a complex thing, and maybe fraught with a little danger if we don't get it right, because we don't have a background and obviously, in pharmaceuticals and medication. But I understand this model. I guess for us, it's more if the client has an issue, or the GP or the pharmacist, and then we become aware of it, and then we step in, and obviously we're happy to facilitate between the client, the GP and the pharmacist, like I said, we have a good lines of communication, (…), so I think that's a good thing, but maybe not suited for us.’ P25
Interprofessional collaboration within the healthcare team	11.1. ‘And I guess collaboration between the hospital pharmacist, community health worker and the community pharmacists that are, you know, seeing the patients more regularly (…), I think would be great. And I think, the community health workers could be involved in medication, I guess, monitoring (…), and help patients. That would be really helpful.’ P10 11.2. ‘And I think the other side is also to do with the transfer of information, is like making sure the patient will understand when the red flags or referral items happen on when to actually not just talk to the community health worker, but actually go back to the GP or doctor.’ P13	11.3. ‘And then the liaison is with the practice. Say they go to see the doctor and their medication gets changed by the doctor, then that has to get feedback into the system so the pharmacist knows to change what's in the people that this kind of thing at a practical level. There's also cases, sometimes when the pharmacists knowledge of particular medications having side effects and things is greater than the GPs. So there's sometimes a case for a little bit of feedback there, in a diplomatic way.’ P27
Support by technology to facilitate CHW role in supporting adherence	12.1. ‘It's a an electronic dosset box which has the medicines. It's filled with the medication, and it prompts the person by an alarm or something, to take their medication. And when the device is turned over, it's deemed the medication has sort of fallen out into their hand, and then it goes back. (…) So that doesn't require an actual worker to be present to prompt medication. So that's a system of adherence that works well for people who are motivated to take their medication but just forgetful. It won't work for someone who wants to throw their medicines down the toilet, because, you know, they'll just do that anyway. So that might be, it may be that the community health worker helps support the implementation of something like this, and then does not need to visit at all times.’ P30	N/A

*Note.* These are all verbatim quotes, and no grammatical corrections have been done.

^a^ Home Medicines Review is a service where a pharmacist visits patients' homes to review the medicines taken by the patient and support the patient to understand the medicines that they take, and make recommendations to general practitioners on the patient's medicines regimen. Reference: Australian Government Department of Health, Disability and Ageing. Home Medicines Review. 2023 [cited 2025 26.12.2025]; Available from: https://www.health.gov.au/our‐work/home‐medicines‐review.

^b^ MedsCheck is a service provided in community pharmacies that gives patients the opportunity to discuss their medicines with a pharmacist, who advises them on the roles of the medicines, how to use and store them, and address any issues. Reference: Australian Government Department of Health, Disability and Ageing. *MedsCheck and Diabetes MedsCheck*. 2023 [cited 2025 26.12.2025]; Available from: https://www.health.gov.au/our‐work/medscheck‐and‐diabetes‐medscheck.

^c^ “My Health Record” is a secure digital record to store health information, available to patients and healthcare providers. Reference: Australian Government Australian Digital Health Agency. My Health Record. 2025 [cited 2025 27.12.2025]; Available from: https://www.digitalhealth.gov.au/initiatives‐and‐programs/my‐health‐record.

^d^ A Webster‐Pak is a type of dose administration aid to help patients manage their medications. It is a blister pack containing the dose of each medication organized by day and time (e.g., morning, noon, evening, night), usually prepared for one week.

### 3.2 Main Themes Identified

A. Theme 1: Perceptions of the model

a. Impact on medication adherence
  Most participants highlighted that nonadherence is prevalent and that there is an important need to monitor medication adherence in a tailored way, as well as support patients to better understand their medications (Quote 1.1., 1.2., 1.3. and 1.4., Table 1). Most pharmacists and CHWs indicated that this CHW‐pharmacist collaborative practice model could improve patient medication adherence monitoring (Quote 1.2., Table 1). A few pharmacists and CHWs explained that this model was currently in practice, where CHWs supported adherence, shared information regarding medication nonadherence with pharmacists and reported side effects.
b. Roles of the CHWs in supporting medication adherence
  Pharmacists reported that CHWs can help patients understand and adhere to their medications (Quote 2.1., Table 1). One CHW explained that they can improve the patient's confidence and understanding regarding medications.
  A CHW working in Australia explained that this model is useful for patients to share their concerns in their own language, when the CHW is from the same cultural background, as CHWs would bridge the gap between pharmacists and patients (Quote 2.2., Table 1).
  One community and academic pharmacist explained that this collaborative practice model makes it clearer as to the benefits of collaborating with CHWs.
c. Improvement of patients' follow‐up by pharmacists and CHWs
  One hospital pharmacist reported that this model could be a good continuity of care for patients, particularly if the patient was cared by the same CHW – in contrast to CHWs ‘being changed all the time’ (P18). A few pharmacists reported that CHWs could help to follow‐up patients in the absence of pharmacists. One community pharmacist said that in Australia, CHWs are usually not directly involved with patients' follow‐ups.
  One pharmacist explained that CHWs would be valuable to follow‐up patients closely and call the doctor in case of a medical emergency (Quote 3.1., Table 1). Another pharmacist explained that the model could work for patients who need regular care and can't see their doctor frequently (e.g., patients on dialysis), so that the CHW can report back to the doctor or the pharmacist in case of a health or medication concern.

B. Theme 2: Challenges to model implementation

a. Overlap of services
  A few pharmacists and one CHW expressed doubts regarding the implementation of the collaborative practice model in Australia. For instance, one academic pharmacist was unsure how this model would be implemented when home medicines review services already exist in Australia, such as Home Medicines Review (HMR) and *MedsCheck*, and the CHWs can already take part in such services. This pharmacist explained that the model might create roles for CHWs which are current roles for pharmacists –the CHWs should not do the HMR instead of the pharmacist (Quote 4.1., Table 1). One pharmacist and one CHW working in New Zealand explained that navigators might not be the ones to take the roles that the collaborative practice model suggests, as there were community home based support services workers (or community health support workers) who currently worked in people's home more regularly than navigators, and monitored patient adherence. Community health support workers report any medication issues to the registered nurse, who would report the issue to the general practitioner (GP) or to the pharmacist directly (Quote 4.2., Table 1).
b. Tailoring the services to the patients' needs
  One CHW reported that the model would need cultural adaptation and consideration of cultural barriers (e.g., in the Pacific culture, grandchild would look after the medications of the elderly) (Quote 5.2., Table 1).
  A pharmacist explained that this model could be applied to a few selected patients (e.g., in case a patient has any change of medication or any new medication). This pharmacist explained that some patients do not necessarily need to see a pharmacist on a regular basis (Quote 5.1., Table 1).
  One CHW working in Australia reported being concerned about the limited time to follow‐up CHWs usually have in Australia with the patients as per their short‐term contracts (e.g., maximum 3 months unless there is a critical reason to continue the patient's follow‐up), which could hamper a good patient follow‐up as suggested by this collaborative model (Quote 5.3., Table 1).
c. CHWs have limited clinical training
  A few pharmacists and CHWs highlighted that CHWs may not have enough training in health or medications (Quote 6.4., 6.5., Table 1), and one CHW working in New Zealand reported that CHWs do not have an understanding of medications to the level that this collaborative practice model requires (Quote 6.6., Table 1). One community pharmacist said that this model would be very challenging for CHWs in case of patients with high needs and critical conditions, considering the limitation of CHWs' clinical training. One CHW said that CHWs are usually not aware of the current medication list for patients and CHWs might say it is not their role to support adherence in the way this model suggests.
  Regarding the phase ‘*Implement plan, follow‐up*’ of the model, two pharmacists explained that CHWs should work within their scope (e.g., CHWs should not be advising on how to manage side effects) and that CHWs should have very clear guidance to guide patients on how to use medicines. There were concerns that CHWs need to have someone to report safety issues to and know when to seek help (Quote 6.1., 6.2., 6.3., Table 1).
d. High need for resources
  Some pharmacists reported that the collaborative practice model would be time‐consuming, and would require pharmacists to be available for it, as pharmacists are already busy and would not have time to be able to do enough follow‐ups with all the patients who go home with CHWs (Quote 7.1., Table 1).
  One hospital pharmacist and one CHW explained that the role they would have within this model would increase their workload in addition to their daily tasks; CHWs might not be able to have time to do their core role if they were to just look at medications as the model suggests (Quote 7.3., Table 1).
  A few pharmacists reported that the model needs a lot of resources to be viable. Indeed, one hospital pharmacist stated that the model is not ready to be implemented in Australia due to the implementation cost (e.g., CHWs' salaries and travel costs to meet the patients in their homes) (Quote 7.2., Table 1). This model would require more funding to be implemented successfully.
e. Challenges to share information between healthcare workers
  One academic pharmacist stated that a barrier to implementing this collaborative practice model could be the confidentiality issues in sharing patient information, as it may not be clear what information the pharmacist can release to CHWs (Quote 8.1., Table 1). Another pharmacist explained that a challenge would be sharing the electronic medication list from a physician to a community pharmacy as they use different systems (Quote 8.2., Table 1). One pharmacist stated that the communication between CHWs and the doctor often happened and might bypass the pharmacist, which is not represented in this model (Quote 8.3., Table 1). One pharmacist explained that CHWs did not usually report back to GPs in Australia and one CHW explained that CHWs did not usually contact the pharmacies at all (Quote 8.4., Table 1).
f. Resistance
  A CHW reported that there might be resistance from healthcare providers if they misunderstand CHWs' roles (Quote 9.2., Table 1). One CHW also stated that most patients get told by their doctors or nurses how to take the medication, instead of the pharmacist. One academic pharmacist explained that there are pros and cons to this model and that the model may be seen as ‘cumbersome’ in the healthcare system, especially regarding CHWs who could do an HMR instead of the pharmacist (see a. Overlap of services) (Quote 9.1., Table 1). Finally, participants noted that this model should be piloted before implementation to understand if it is working and what to improve.

C. Theme 3: Facilitators for model implementation
  Most pharmacists and CHWs working in Australia and New Zealand said that the model could be implemented in their own country. They provided some strategies to help the implementation of the model.

a. Clarification of roles
  A few pharmacists reported that increasing the clarity of the roles would help the implementation of this practice model, as there might be overlap of roles (Quote 10.1., Table 1). One academic pharmacist said that CHWs could be seen as the ‘agent of the patient’ (P13). One hospital pharmacist explained that this model is ‘macro’ (P18) and it would benefit from details about what to expect regarding CHWs' roles, as this model would be the start of consolidating CHWs' roles. One pharmacist working in New Zealand said that this model could empower CHWs so that they are more involved and have a better relationship with patients.
  A few pharmacists suggested to provide training and education to upskill CHWs, and that CHWs should be under close supervision (Quote 10.2., Table 1), to make sure that information is being clearly communicated to the patient. One CHW working in New Zealand explained that he/she does not want to get involved with people's medication, as he/she feels they do not have the required expertise for this role (Quote 10.3., Table 1). One CHW suggested letting clinicians know what the model entails to avoid resistance to CHWs' roles (Quote 9.2., Table 1).
  Additionally, one hospital pharmacist reported that if a position was available for a pharmacist to take the roles that this model of care requires, it would take stress off doctors (including GPs) as they would be able to follow‐up with patients and CHWs (Quote 7.1., Table 1).
b. Interprofessional collaboration within the healthcare team
  Participants highlighted the need for collaboration with GPs and hospital pharmacists within this practice model; one hospital pharmacist highlighted that the collaboration between CHWs, community pharmacists and hospital pharmacists would also be valuable in this practice model (Quote 11.1., Table 1). Another pharmacist explained that the patient needed to be aware of red flags to know when to go to the GP and not only report to the CHW (Quote 11.2., Table 1).
  Regarding the phase ‘*Pharmacist collaborate with CHW to develop action plan to address adherence barriers for CHW to implement*’, one pharmacist said that the medication plan needed to be in consultation with the physician. Another pharmacist explained that the CHW should be involved early in the process of building the medication plan. One CHW reported that pharmacists should give feedback to the GP regarding changes in a patient's medications or side effects experienced by the patient (Quote 11.3., Table 1).
c. Support by technology to facilitate CHW role in supporting adherence
  One pharmacist working in New Zealand explained that the CHWs can help support the implementation of an electronic carousel device to support medication adherence in case of patient's medication forgetfulness (Quote 12.1., Table 1).

## 4 Discussion

This study explored the perceptions of CHWs and pharmacists working in Australia and New Zealand on a collaborative CHW‐pharmacist practice model developed in the USA [25]. This model involves CHWs collecting information on medication lists and medication adherence barriers, sharing information with the pharmacist to develop an action plan to address adherence barriers, CHW helping to implement the plan with the patient and follow‐up to assess resolution of adherence barriers and presence of new ones. The opinions of participants were across three main themes: (1) perceptions of the model, (2) challenges to model implementation and (3) facilitators for model implementation.

Most participants recognised the potential of the model to support medication adherence and appreciated the role of the CHW in bridging the cultural gap with patients, supporting patients' medication understanding and follow up with patients. However, they reported challenges to the implementation of the model in Australia and New Zealand, such as the concern of overlap of services or overtaking of roles, cultural considerations, CHWs' limited clinical training, need for resources and the challenge of sharing information with the healthcare team members. Facilitators for model implementation included clarification of roles, training for CHWs, and fostering collaboration with other members of the healthcare team besides pharmacists and CHWs (e.g., hospital pharmacists, GPs).

The current model suggests a synergic collaboration between pharmacists and CHWs, where pharmacists have clinical roles and CHWs are facilitators in bridging the cultural gap and helping patients implement the medication plan.

For the model to be implemented in Australia and New Zealand, it needs to be adapted to the local contexts and tailored to the patients' needs. The Australian and New Zealand healthcare systems are both universal, supported by the Government, with a mix of public and private funding schemes [45, 46]. They differ from the healthcare system in the USA, which is mostly private insurance based. In Australia and New Zealand, as the healthcare systems of both countries are similar, the implementation strategies can be easily translated from one country to another. Both healthcare systems face common challenges, such as health disparities for their indigenous populations (i.e., Aboriginal and Torres Strait Islander and Māori), the aging populations and staff shortages [45, 46].

Pharmacists' roles are quite similar in the USA, Australia and New Zealand, especially regarding medication adherence support and quality use of medicines. However, CHWs are more integrated into healthcare teams in the USA, where they have worked in diverse settings for more than 70 years and are recognised as essential healthcare workforce [47]. The implementation of such a model can be more challenging in Australia and New Zealand, where the CHW position is less recognised and CHWs are less embedded into healthcare teams [48, 49].

CHWs' roles, responsibilities and training vary across countries and depend on the needs of the local communities [50, 51, 52]. For instance, a health navigator programme exists on the West Coast of New Zealand [53], where navigators may already be involved in sharing information on patient medication adherence with pharmacists. In Australia, CHWs may already participate in home medicines reviews with pharmacists and patients [54].

Evaluating the pharmaceutical services already in place to support adherence and assessing how a CHW could be integrated in these services would be valuable to avoid duplication of services. However, this model may also be viewed as a way of providing current pharmacy services where pharmacists are supported by CHWs. Additionally, this model demonstrates the evolution of pharmacy services, where the services which have been traditionally provided by pharmacists alone, can now be delivered by a team, where CHWs have been integrated into the pharmacy services [55].

To successfully implement this model in Australia and New Zealand, it is essential to enhance the training on medication adherence support for CHWs. In our study, CHWs expressed low confidence in their medication knowledge, and pharmacists were concerned about CHWs having roles beyond their scope of practice. In the USA, 109 CHWs were trained on medication adherence support for patients with hypertension [56]. The programme, led by clinical pharmacists, consisted of 6 h of pre‐recorded lectures before a 2‐day live training (including real‐life scenario case studies). It showed significant improvement in CHWs' knowledge and confidence to support medication adherence [56]. Of note, the training was also delivered to 19 pharmacy technicians and also showed similar levels of improvement in knowledge and self‐efficacy [57]. This training could be implemented in Australia and New Zealand as a facilitator to the implementation of the CHW‐pharmacist practice model to consolidate CHWs' roles to support medication adherence.

Additionally, the model should be implemented with clear definitions of CHWs' and pharmacists' roles and guidelines on collaborative practices (e.g., how, when, where to share patient information, frequency of meetings). The clarification of roles may also help prevent resistance from other healthcare workers and avoid encroaching on their roles [58].

The next steps to build and implement a CHW‐pharmacist collaborative practice model in Australia and New Zealand are to conduct research on (1) the unmet needs of the local population in terms of medication adherence, (2) collecting perspectives of this model from people from culturally and linguistically diverse populations, (3) co‐designing the adaptations of the model with consumer involvement, especially with local cultural considerations, (4) developing implementation strategies to integrate this model in the local context, (5) piloting the model to ensure integration in the local context and that it corresponds to the local population needs (i.e., the model may be first tested in specific vulnerable populations, e.g., patients with polypharmacy, patients after hospital discharge, migrants) and (6) raise awareness of the model before implementation to avoid resistance in the interprofessional healthcare team.

Government and policy makers (e.g., particularly those working locally such as local health districts and primary health networks) should be aware of the potential roles that CHWs could take to support patient adherence in their communities in collaboration with the pharmacists, in order to allow resources and funding for the research and the development of CHW and CHW‐pharmacist collaborative programmes across Australia and New Zealand.

The roles of healthcare providers are also affected by workforce capacity, especially in rural and remote areas of Australia and New Zealand. Integrating CHWs in healthcare teams alongside pharmacists, can help support patient care, especially in the context of growing workforce shortages. For instance, in rural and remote areas, CHWs embedded within their communities can work closely with patients in the absence of pharmacists, and report any health concerns to pharmacists via phone, video calls or other remote technologies.

Some limitations are to be acknowledged. Firstly, the findings should not be generalised beyond the group of participants interviewed, i.e., CHWs and pharmacists working in Australia and New Zealand, where participants were mostly recruited through the researchers' professional networks. CHWs working in hospital settings were not interviewed in this study. While they may have provided different viewpoints compared to CHWs in community settings, we did interview CHWs who worked at the transition of care between hospital and community. Moreover, the CHWs interviewed worked in diverse community settings, which added further perspectives to the findings. Secondly, the participants worked in urban areas (e.g., metropolitan areas or small towns); we did not interview participants working in rural or remote areas. The practice location may have an impact on workforce capacity and roles, and viewpoints may vary. Thirdly, social desirability bias —where participants provide answers that they perceive to be more acceptable by the researcher than their own ideas and opinions [59], cannot be excluded.

## 5 Conclusion

Most CHWs and pharmacists working in Australia and New Zealand interviewed recognised the potential of the CHW‐pharmacist collaborative practice model to support patient medication adherence developed in the USA. This model consisted of CHWs collecting information on medication adherence barriers, sharing information with the pharmacist to develop an action plan to address them, and CHWs following up to implement the plan with the patient.

Pharmacists and CHWs appreciated the role of CHWs in complementing pharmacists' roles by bridging the cultural gap with the patient, supporting medication understanding and following up with the patient. Challenges to model implementation in Australia and New Zealand included concern of overlap of services, CHWs' limited clinical training, need for cultural adaptations, resources and the process of sharing information with the healthcare team members. Facilitators for model implementation included clarification of roles, training for CHWs, and fostering collaboration with the other members of the healthcare team besides the pharmacists and the CHWs. The limited clinical skills of the CHWs regarding medication management could be resolved by delivering specific medication management education and training to CHWs.

Developing national guidelines on the CHW‐pharmacist collaboration to support medication adherence can facilitate the implementation of this collaborative CHW‐pharmacist practice model into healthcare systems in Australia and New Zealand.

## Author Contributions

**Carole Bandiera:** conceptualisation, investigation, funding acquisition, writing – original draft, methodology, visualisation, writing – review and editing, formal analysis, project administration, data curation. **Sabuj Kanti Mistry**, **Elizabeth Harris**, and **Mark F. Harris:** conceptualisation, investigation, methodology, validation, visualisation, writing – review and editing, formal analysis. **Parisa Aslani:** conceptualisation, investigation, methodology, validation, visualisation, writing – review and editing, formal analysis, project administration, data curation, supervision, resources.

## Ethics Statement

The study was conducted in accordance with the declaration of Helsinki and was approved by the Human Research Ethics Committee of The University of Sydney (2024/HE000334).

## Consent

Written consent was obtained before the start of data collection.

## Conflicts of Interest

The authors declare no conflicts of interest.

## Supporting information


**Supporting Material 1:** COREQ checklist.

**Supporting Material 2:** Interview guide for pharmacists.

**Supporting Material 3:** Interview guide for CHWs.

**Supporting Material 4:** Demographic data of participants.

## Data Availability

The datasets generated and analysed during the current study are not publicly available due to the approval received from the institutional Human Research Ethics Committee.
