# Supplementary material for: Community Health Workers' and Pharmacists' Perspectives of a CHW‐Pharmacist Collaboration Model to Support Medication Adherence
Source: Health Expect. 2026 Mar 16;29(2):e70630. doi: 10.1111/hex.70630 (PMC13080894; doi:10.1111/hex.70630)
Supplement: Supplementary file 1 — Supporting Material 1: COREQ checklist. [file HEX-29-e70630-s003.docx]

**Consolidated criteria for reporting qualitative studies (COREQ) 32-item checklist**

Developed from: Allison Tong, Peter Sainsbury, Jonathan Craig, Consolidated criteria for reporting qualitative research (COREQ): a 32-item checklist for interviews and focus groups, International Journal for Quality in Health Care, Volume 19, Issue 6, December 2007, Pages 349–357, <https://doi.org/10.1093/intqhc/mzm042>

| **Item No** | | **Guide Questions/Description** | **Answers** |  |
| --- | --- | --- | --- | --- |
| **Domain 1: Research team and reflexivity** | | | |  |
| **Personal Characteristics** | | | |  |
| 1. Interviewer/facilitator | | Which author/s conducted the interview or focus group? | The first author, Carole Bandiera, conducted all interviews. |  |
| 2. Credentials | | What were the researcher’s credentials? E.g., PhD, MD | Dr Carole Bandiera, PhD  Dr Sabuj Kanti Mistry, PhD  Prof Elizabeth Harris, PhD  Prof Mark F. Harris, PhD  Prof Parisa Aslani, PhD |  |
| 3. Occupation | | What was their occupation at the time of the study? | Dr Carole Bandiera is a postdoctoral research fellow at the University of Sydney in the School of Pharmacy.  Dr Sabuj Kanti Mistry is a lecturer at the University of New South Wales, School of Population Health.  Adjunct associate Professor Elizabeth Harris, retired, International Centre for Future Health Systems, University of New South Wales.  Emeritus Professor Mark Harris, retired, International Centre for Future Health Systems, University of New South Wales.  Professor Parisa Aslani is an academic at the University of Sydney in the School of Pharmacy. |  |
| 4. Gender | | Was the researcher male or female? | Carole Bandiera (CB) is a female. |  |
| 5. Experience and training | | What experience or training did the researcher have? | CB was trained and had robust experience in qualitative research. |  |
| **Relationship with participants** | | | |  |
| 6. Relationship established | | Was a relationship established prior to study commencement? | Face to face interviews  Most of the participants were contacted by email prior to the interview. Most of the participants interviewed face-to-face learned about the study through email. No formal relationship was established prior to data collection.  Online and phone interviews  Most of the participants interviewed online or by phone learned about the study through email or electronic advertising. No formal relationship was established with these participants prior  to the study. |  |
| 7. Participant knowledge of the interviewer | | What did the participants know about the researcher? e.g. personal goals, reasons for doing the research? | The participants knew the information provided on the study flyer and the participant information statement, i.e., that CB was a postdoctoral research fellow at the University of Sydney, School of Pharmacy. CB explained that she was a pharmacist by training and explained the aim of the research, i.e., to better understand how pharmacists and community health workers can work together. |  |
| 8. Interviewer characteristics | | What characteristics were reported about the interviewer/facilitator? e.g. Bias, assumptions, reasons and interests in the research topic | CB, who conducted the reflexive thematic analysis, is a pharmacist by training, which might have influenced the interpretation of the findings towards a better understanding of the perceptions, challenges and facilitators for pharmacists within the CHW-pharmacist collaboration. To limit potential data interpretation, the coding tree was discussed and reviewed until consensus achieved with SKM, who is not a pharmacist by training, and PA. The codes were then reviewed by EH and MH who are not pharmacists by training. All researchers were trained and had extensive experience in qualitative research. |  |
| **Domain 2: study design** | | |  |  |
| **Theoretical framework** | | |  |  |
| 9. Methodological orientation and Theory | What methodological orientation was stated to underpin the study? e.g. grounded theory, discourse analysis, ethnography, phenomenology, content analysis | The transcripts were de-identified and analysed following Braun and Clarke’s work through a reflexive thematic analysis and an inductive process, acknowledging the researcher’s subjectivity[1-3]:  We created the topic summaries by: 1) reading of raw data, i.e. transcribed verbatims, 2) iterative identification of text segments in line with the study aims and relevant content, 3) identification of text segments to create and develop codes and sub-codes, 4) reduction of similar or redundant codes and sub-codes, 5) iterative process of refining the codes as the study findings evolved, 6) creation of a coding tree that integrates important codes into categories and main themes and subthemes, and 7) interpretation of the theme development, acknowledging reflexivity and subjectivity. |  |  |
| **Participant selection** | | |  |  |
| 10. Sampling | How were participants selected? e.g., purposive, convenience, consecutive, snowball | Different approaches were used to recruit the participants from Australia and New-Zealand: i) potential participants were identified through the professional network of authors, ii) the study flyer was displayed and advertised at the University of Sydney and through the School of Pharmacy’s newsletter, iii) the study flyer was advertised through an Australian professional association’s newsletter sent to pharmacists, iv) some study participants promoted the study in their professional network, v) the study was promoted on the professional platform LinkedIn (LinkedIn Corporation) and on closed pharmacist groups on Facebook (Meta Platforms, Inc.).  Our sample is not a random sample of the population but was mostly restricted to the researchers’ and health professionals’ networks through the snowballing recruitment approach. |  |  |
| 11. Method of approach | How were participants approached? e.g., face-to-face, telephone, mail, email | The initial contact with potential participants to introduce the study was made via email, over the phone or video-call by CB, along with sending the participant information statement (for more details, see 10. above). Written informed consent was obtained before data collection. |  |  |
| 12. Sample size | How many participants were in the study? | Twenty-nine participants agreed to participate, including 16 pharmacists and 13 community health workers (CHWs). |  |  |
| 13. Non-participation | How many people refused to participate or dropped out? Reasons? | In total, 34 eligible participants were approached (18 pharmacists and 16 CHWs) and 29 participants (85%) agreed to participate, including 16/18 pharmacists (89%) and 13/16 CHWs (81%).  Of those who did not participate, some were not interested in taking part in the research while others did not respond to further communication about the study.  None of the participants dropped out. |  |  |
| **Setting** | | |  |  |
| 14. Setting of data collection | Where was the data collected? e.g., home, clinic, workplace | Interviews were conducted either online using Zoom (Zoom Video Communications, Inc.) or Teams videoconferencing (Microsoft Corporation), or in-person at the participant’s place of work (e.g., the University of Sydney, the health organization), at an agreed time and in an appropriate place ensuring participant’s privacy and confidentiality. |  |  |
| 15. Presence of non-participants | Was anyone else present besides the participants and researchers? | No one else was present besides the participant and the researcher, CB. |  |  |
| 16. Description of sample | What are the important characteristics of the sample? e.g. demographic data, date | The eligibility criteria were: i) a CHW, the CHW supervisor or a pharmacist who has been working in health services for at least 6 months in Australia or New Zealand and ii) speaks English and does not require an interpreter.  There were 10 female pharmacists and 10 female CHWs. The median pharmacist age was 32 years old and the median CHWs age was 50 years old (see Table 1).  Semi-structured interviews were conducted by CB between July 2024 and February 2025. |  |  |
| **Data collection** | | |  | No |
| 17. Interview guide | Were questions, prompts, guides provided by the authors? Was it pilot tested? | The interview guides were developed by the authors and are presented in Supplementary Materials 2 and 3.  Prior to data collection, both pharmacist and CHW interview guides were piloted with three academic pharmacists and three CHWs. |  |  |
| 18. Repeat interviews | Were repeat interviews carried out? If yes, how many? | No repeat interviews were carried out. |  |  |
| 19. Audio/visual recording | Did the research use audio or visual recording to collect the data? | The interviews were audio-recorded with recorders or using Zoom (Zoom Video Communications, Inc.) or Teams videoconferencing (Microsoft Corporation). |  |  |
| 20. Field notes | Were field notes made during and/or after the interview or focus group? | CB collected field notes during the interviews. |  |  |
| 21. Duration | What was the duration of the interviews or focus group? | The median duration of the interviews was 30 minutes (IQR 26; 34) with pharmacists and 31 minutes (IQR 27; 36) with CHWs |  |  |
| 22. Data saturation | Was data saturation discussed? | Data saturation was reached after 12/16 interviews with pharmacists and 10/13 interviews with CHWs. However, further interviews were conducted to confirm data saturation. |  |  |
| 23. Transcripts returned | Were transcripts returned to participants for comment and/or correction? | The transcription and coding of interviews were done alongside data collection. The interviews were transcribed verbatim and any identifying information was removed from the transcripts. All participants were offered to review their interview transcript, two pharmacists and seven CHWs reviewed their transcript and no significant changes or comments were made. |  |  |
| **Domain 3: analysis and findings** | | |  |  |
| **Data analysis** | | |  |  |
| 24. Number of data coders | How many data coders coded the data? | CB, who conducted the reflexive thematic analysis, is a pharmacist by training, which might have influenced the interpretation of the findings towards a better understanding of the perceptions, challenges and facilitators for pharmacists within the CHW-pharmacist collaboration. To limit potential data interpretation, the coding tree was discussed and reviewed until consensus achieved with SKM, who is not a pharmacist by training, and PA. The codes were then reviewed by MH and EH who are not pharmacists by training. All researchers were trained and had robust experience in qualitative research. |  |  |
| 25. Description of the coding tree | Did authors provide a description of the coding tree? | Participants expressed their opinions about the CHW-pharmacist collaborative practice model around three main themes: 1) perceptions of the model, 2) challenges to the model implementation, 3) facilitators for the model implementation. Figure 2 summarizes the key themes and subthemes. |  |  |
| 26. Derivation of themes | Were themes identified in advance or derived from the data? | Themes were derived from the data. |  |  |
| 27. Software | What software, if applicable, was used to manage the data? | The interviews were coded using the qualitative data analysis software NVivo version 14 (QSR International Pty Ltd).  All study forms (e.g., participant consent form, demographic data and receipt of the electronic voucher) were filled-in by participants in hard copy or online — using Research Electronic Data Capture (RedCap, Vanderbilt University), a secure, web-based software platform designed to support data capture for research studies[4]. |  |  |
| 28. Participant checking | Did participants provide feedback on the findings? | A summary of the results was sent to all participants and only one person provided feedback, which did not have any impact on the study findings and the reporting of the findings. |  |  |
| **Reporting** | | |  |  |
| 29. Quotations presented | Were participant quotations presented to illustrate the themes/findings? Was each quotation identified? e.g., participant number | The results have been described narratively along with citations of relevant quotes, each quote was identified by a number along with the participant identification number (see Table 1). |  |  |
| 30. Data and findings consistent | Was there consistency between the data presented and the findings? | The quotes from the data were carefully chosen to illustrate the themes and subthemes. We believe that there is consistency between the data presented and the findings of the research. |  |  |
| 31. Clarity of major themes | Were major themes clearly presented in the findings? | Major themes are clearly presented in the text and illustrated in Figure 2. |  |  |
| 32. Clarity of minor themes | Is there a description of diverse cases or a discussion of minor themes? | For each major themes, subthemes were described and discussed in the text and illustrated in Figure 2. |  |  |

References:

1. Braun, V. and V. Clarke, *Using thematic analysis in psychology.* Qualitative Research in Psychology, 2006. **3**(2): p. 77-101.

2. Braun, V. and V. Clarke, *Toward good practice in thematic analysis: Avoiding common problems and be(com)ing a knowing researcher.* Int J Transgend Health, 2023. **24**(1): p. 1-6.

3. Braun, V. and V. Clarke, *One size fits all? What counts as quality practice in (reflexive) thematic analysis?* Qualitative Research in Psychology, 2020. **18**(3): p. 328-352.

4. Harris, P.A., R. Taylor, R. Thielke, J. Payne, N. Gonzalez, and J.G. Conde, *Research electronic data capture (REDCap)--a metadata-driven methodology and workflow process for providing translational research informatics support.* J Biomed Inform, 2009. **42**(2): p. 377-81.
