# Supplementary material for: Community Health Workers' and Pharmacists' Perspectives of a CHW‐Pharmacist Collaboration Model to Support Medication Adherence
Source: Health Expect. 2026 Mar 16;29(2):e70630. doi: 10.1111/hex.70630 (PMC13080894; doi:10.1111/hex.70630)
Supplement: Supplementary file 4 — Supporting Material 4: Demographic data of participants. [file HEX-29-e70630-s001.docx]

**Participants' demographic data**

| Demographic data (n=29) | **Pharmacists (n=16)** | | **CHWs (n=13)** | |
| --- | --- | --- | --- | --- |
| **Self-reported professional position(s), n (%)** | | | | |
|  | Hospital pharm.  Academic pharm.  Community & academic pharm.  Community & hospital pharm.  Community pharm.  Community and research-based pharm.  Hospital and research-based pharm.  Academic & hospital pharm. | 5 (31)  2 (13)  2 (13)  2 (13)  2 (13)  1 (6)  1 (6)  1 (6) | Health navigator  Community health navigator  Cultural support worker  Bilingual community educator  Psychosocial support worker  Supervisor health navigator  Smokefree services coordinator | 4 (31)  2 (15)  2 (15)  2 (15)  1 (8)  1 (8)  1 (8) |
| **Self-identified gender, n (%)** | | | | |
| Female  Male | 10 (62)  6 (38) | | 10 (77)  3 (23) | |
| **Years of work experience in health, median, (IQR)** | | | | |
|  | 9 (6-19) | | 8 (3-17) | |
| **Self-identified cultural background in the checklist provided*, n (%)** | | | | |
|  | Oceanian  North-West European  South-East Asian  Other**, as defined by participants | 2 (13)  1 (6)  5 (31)  8 (50) | Oceanian  Other***, as defined by participants | 2 (15)  11 (85) |
| **Age in years, median (IQR)** | | | | |
|  | 32 (30-43) | | 50 (40-59) | |
| **Place of work, n (%)** | | | | |
| Australia  New Zealand | 12 (75)  4 (25) | | 7 (54)  6 (46) | |

Abbreviations: IQR, interquartile range; pharm., pharmacist(s).

*Some of the participants reported several cultural backgrounds.

**The “Other” category includes Australian, Aboriginal and Torres Strait Islander, Caucasian, New Zealander, Māori

***The “Other” category includes New Zealander, Bangladeshi, Chinese, Australian, Eastern Asian, Indian, Northern, English, Māori, Irish, Samoa, Pacific islander, Syrian
